# Impact of pesticides use in agriculture: their benefits and hazards

**DOI:** 10.2478/v10102-009-0001-7

**Published:** 2009-03

**Authors:** Md. Wasim Aktar, Dwaipayan Sengupta, Ashim Chowdhury

**Affiliations:** 1 Pesticide Residue Laboratory, Department of Agricultural Chemicals, Bidhan Chandra Krishi Viswavidyalaya, Mohanpur, Nadia, West Bengal, India; 2 Department of Agricultural Chemistry and Soil Science, Institute of Agricultural Science, University of Calcutta, Kolkata, West Bengal, India

**Keywords:** pesticides, India, quality of food, environment

## Introduction

The term pesticide covers a wide range of compounds including insecticides, fungicides, herbicides, rodenticides, molluscicides, nematicides, plant growth regulators and others. Among these, organochlorine (OC) insecticides, used successfully in controlling a number of diseases, such as malaria and typhus, were banned or restricted after the 1960s in most of the technologically advanced countries. The introduction of other synthetic insecticides – organophosphate (OP) insecticides in the 1960s, carbamates in 1970s and pyrethroids in 1980s and the introduction of herbicides and fungicides in the 1970s–1980s contributed greatly to pest control and agricultural output. Ideally a pesticide must be lethal to the targeted pests, but not to non-target species, including man. Unfortunately, this is not the case, so the controversy of use and abuse of pesticides has surfaced. The rampant use of these chemicals, under the adage, “if little is good, a lot more will be better” has played havoc with human and other life forms.

### Production and usage of pesticides in India

The production of pesticides started in India in 1952 with the establishment of a plant for the production of BHC near Calcutta, and India is now the second largest manufacturer of pesticides in Asia after China and ranks twelfth globally (Mathur, 1999). There has been a steady growth in the production of technical grade pesticides in India, from 5,000 metric tons in 1958 to 102,240 metric tons in 1998. In 1996–97 the demand for pesticides in terms of value was estimated to be around Rs. 22 billion (USD 0.5 billion), which is about 2% of the total world market.

**Figure 1:**
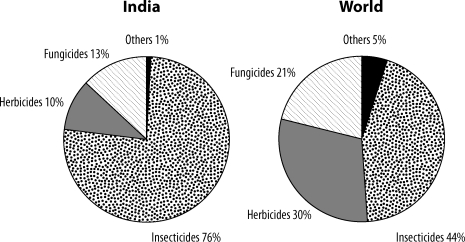
Consumption pattern of pesticides.

The pattern of pesticide usage in India is different from that for the world in general. As can be seen in Figure 1, in India 76% of the pesticide used is insecticide, as against 44% globally (Mathur, 1999). The use of herbicides and fungicides is correspondingly less heavy. The main use of pesticides in India is for cotton crops (45%), followed by paddy and wheat.

## Benefits of pesticides

The primary benefits are the consequences of the pesticides' effects – the direct gains expected from their use. For example the effect of killing caterpillars feeding on the crop brings the primary benefit of higher yields and better quality of cabbage. The three main effects result in 26 primary benefits ranging from protection of recreational turf to saved human lives. The secondary benefits are the less immediate or less obvious benefits that result from the primary benefits. They may be subtle, less intuitively obvious, or of longer term. It follows that for secondary benefits it is therefore more difficult to establish cause and effect, but nevertheless they can be powerful justifications for pesticide use. For example the higher cabbage yield might bring additional revenue that could be put towards children's education or medical care, leading to a healthier, better educated population. There are various secondary benefits identified, ranging from fitter people to conserved biodiversity.

### Improving productivity

Tremendous benefits have been derived from the use of pesticides in forestry, public health and the domestic sphere – and, of course, in agriculture, a sector upon which the Indian economy is largely dependent. Food grain production, which stood at a mere 50 million tons in 1948–49, had increased almost fourfold to 198 million tons by the end of 1996–97 from an estimated 169 million hectares of permanently cropped land. This result has been achieved by the use of high-yield varieties of seeds, advanced irrigation technologies and agricultural chemicals (Employment Information: Indian Labour Statistics, 1994). Similarly outputs and productivity have increased dramatically in most countries, for example wheat yields in the United Kingdom, corn yields in the USA. Increases in productivity have been due to several factors including use of fertiliser, better varieties and use of machinery. Pesticides have been an integral part of the process by reducing losses from the weeds, diseases and insect pests that can markedly reduce the amount of harvestable produce. Warren (1998) also drew attention to the spectacular increases in crop yields in the United States in the twentieth century. Webster *et al.* (1999) stated that “considerable economic losses” would be suffered without pesticide use and quantified the significant increases in yield and economic margin that result from pesticide use. Moreover, in the environment most pesticides undergo photochemical transformation to produce metabolites which are relatively non-toxic to both human beings and the environment (Kole *et al*., 1999).

### Protection of crop losses/yield reduction

In medium land, rice even under puddle conditions during the critical period warranted an effective and economic weed control practice to prevent reduction in rice yield due to weeds that ranged from 28 to 48%, based on comparisons that included control (weedy) plots (Behera and Singh, 1999). Weeds reduce yield of dry land crops (Behera and Singh, 1999) by 37–79%. Severe infestation of weeds, particularly in the early stage of crop establishment, ultimately accounts for a yield reduction of 40%. Herbicides provided both an economic and labour benefit.

### Vector disease control

Vector-borne diseases are most effectively tackled by killing the vectors. Insecticides are often the only practical way to control the insects that spread deadly diseases such as malaria, resulting in an estimated 5000 deaths each day (Ross, 2005). In 2004, Bhatia wrote that malaria is one of the leading causes of morbidity and mortality in the developing world and a major public health problem in India. Disease control strategies are crucially important also for livestock.

### Quality of food

In countries of the first world, it has been observed that a diet containing fresh fruit and vegetables far outweigh potential risks from eating very low residues of pesticides in crops (Brown, 2004). Increasing evidence (Dietary Guidelines, 2005) shows that eating fruit and vegetables regularly reduces the risk of many cancers, high blood pressure, heart disease, diabetes, stroke, and other chronic diseases.

Lewis *et al.* (2005) discussed the nutritional properties of apples and blueberries in the US diet and concluded that their high concentrations of antioxidants act as protectants against cancer and heart disease. Lewis attributed doubling in wild blueberry production and subsequent increases in consumption chiefly to herbicide use that improved weed control.

### Other areas – transport, sport complex, building

The transport sector makes extensive use of pesticides, particularly herbicides. Herbicides and insecticides are used to maintain the turf on sports pitches, cricket grounds and golf courses. Insecticides protect buildings and other wooden structures from damage by termites and woodboring insects.

## Hazards of pesticides

### Direct impact on humans

If the credits of pesticides include enhanced economic potential in terms of increased production of food and fibre, and amelioration of vector-borne diseases, then their debits have resulted in serious health implications to man and his environment. There is now overwhelming evidence that some of these chemicals do pose a potential risk to humans and other life forms and unwanted side effects to the environment (Forget, 1993; Igbedioh, 1991; Jeyaratnam, 1981). No segment of the population is completely protected against exposure to pesticides and the potentially serious health effects, though a disproportionate burden, is shouldered by the people of developing countries and by high risk groups in each country (WHO, 1990). The world-wide deaths and chronic diseases due to pesticide poisoning number about 1 million per year (Environews Forum, 1999).

The high risk groups exposed to pesticides include production workers, formulators, sprayers, mixers, loaders and agricultural farm workers. During manufacture and formulation, the possibility of hazards may be higher because the processes involved are not risk free. In industrial settings, workers are at increased risk since they handle various toxic chemicals including pesticides, raw materials, toxic solvents and inert carriers.

OC compounds could pollute the tissues of virtually every life form on the earth, the air, the lakes and the oceans, the fishes that live in them and the birds that feed on the fishes (Hurley *et al*., 1998). The US National Academy of Sciences stated that the DDT metabolite DDE causes eggshell thinning and that the bald eagle population in the United States declined primarily because of exposure to DDT and its metabolites (Liroff, 2000). Certain environmental chemicals, including pesticides termed as endocrine disruptors, are known to elicit their adverse effects by mimicking or antagonising natural hormones in the body and it has been postulated that their long-term, low-dose exposure is increasingly linked to human health effects such as immune suppression, hormone disruption, diminished intelligence, reproductive abnormalities and cancer (Brouwer *et al*., 1999; Crisp *et al*., 1998; Hurley *et al*., 1998)

A study on workers (N=356) in four units manufacturing HCH in India revealed neurological symptoms (21%) which were related to the intensity of exposure (Nigam et al., 1993). The magnitude of the toxicity risk involved in the spraying of methomyl, a carbamate insecticide, in field conditions was assessed by the National Institute of Occupational Health (NIOH) (Saiyed *et al.*, 1992). Significant changes were noticed in the ECG, the serum LDH levels, and cholinesterase (ChE) activities in the spraymen, indicating cardiotoxic effects of methomyl. Observations confined to health surveillance in male formulators engaged in production of dust and liquid formulations of various pesticides (malathion, methyl parathion, DDT and lindane) in industrial settings of the unorganised sector revealed a high occurrence of generalised symptoms (headache, nausea, vomiting, fatigue, irritation of skin and eyes) besides psychological, neurological, cardiorespiratory and gastrointestinal symptoms coupled with low plasma ChE activity (Gupta *et al*., 1984).

Data on reproductive toxicity were collected from 1,106 couples when the males were associated with the spraying of pesticides (OC, OP and carbamates) in cotton fields (Rupa et al., 1991).A study in malaria spraymen was initiated to evaluate the effects of a short-term (16 week) exposure in workers (N=216) spraying HCH in field conditions (Gupta *et al*., 1982).

A study on those affected in the Seveso diaster of 1976 in Italy during the production of 2,4,5 T, a herbicide, concluded that chloracne (nearly 200 cases with a definite exposure dependence) was the only effect established with certainty as a result of dioxin formation (Pier *et al*., 1998). Early health investigations including liver function, immune function, neurologic impairment, and reproductive effects yielded inconclusive results. An excess mortality from cardiovascular and respiratory diseases was uncovered, possibly related to the psychosocial consequences of the accident in addition to the chemical contamination. An excess of diabetes cases was also found. Results of cancer incidence and mortality follow-up showed an increased occurrence of cancer of the gastrointestinal sites and of the lymphatic and haematopoietic tissue. Results cannot be viewed as conclusive, however, because of various limitations: few individual exposure data, short latency period, and small population size for certain cancer types. A similar study in 2001 observed no increase in all-cause and all-cancer mortality. However, the results support the notion that dioxin is carcinogenic to humans and corroborate the hypotheses of its association with cardiovascular- and endocrine-related effects (Pier *et al*., 2001). During the Vietnam War, United States military forces sprayed nearly 19 million gallons of herbicide on approximately 3.6 million acres of Vietnamese and Laotian land to remove forest cover, destroy crops, and clear vegetation from the perimeters of US bases. This effort, known as Operation Ranch Hand, lasted from 1962 to 1971. Various herbicide formulations were used, but most were mixtures of the phenoxy herbicides 2,4-dichlorophenoxyacetic acid (2,4-D) and 2,4,5-trichlorophenoxyacetic acid (2,4,5-T). Approximately 3 million Americans served in the armed forces in Vietnam during the Vietnam War. Some of them (as well as some Vietnamese combatants and civilians, and members of the armed forces of other nations) were exposed to defoliant mixtures, including Agent Orange. There was evidence on cancer risk of Vietnam veterans, workers occupationally exposed to herbicides or dioxins (since dioxins contaminated the herbicide mixtures used in Vietnam), and of the Vietnamese population (Frumkin, 2003).

### Impact through food commodities

For determining the extent of pesticide contamination in the food stuffs, programs entitled ‘Monitoring of Pesticide Residues in Products of Plant Origin in the European Union’ started to be established in the European Union since 1996. In 1996, seven pesticides (acephate, chlopyriphos, chlopyriphos-methyl, methamidophos, iprodione, procymidone and chlorothalonil) and two groups of pesticides (benomyl group and maneb group, i.e. dithiocarbamates) were analysed in apples, tomatoes, lettuce, strawberries and grapes. An average of about 9 700 samples has been analysed for each pesticide or pesticide group. For each pesticide or pesticide group, 5.2% of the samples were found to contain residues and 0.31% had residues higher than the respective MRL for that specific pesticide. Lettuce was the crop with the highest number of positive results, with residue levels exceeding the MRLs more frequently than in any of the other crops investigated. The highest value found in 1996 was for a compound of the maneb group in lettuce which corresponded to a mancozeb residue of 118 mg/kg. In 1997, 13 pesticides (acephate, carbendazin, chlorothalonil, chlopyriphos, DDT, diazinon, endosulfan, methamidophos, iprodione, metalaxyl, methidathion, thiabendazole, triazophos) were assessed in five commodities (mandarins, pears, bananas,beans, and potatoes). Some 6 000 samples were analysed. Residues of chlorpyriphos exceeded MRLs most often (0.24%), followed by methamidophos (0.18%), and iprodione (0.13%). With regard to the commodities investigated, around 34% contained pesticide residues at or below the MRL, and 1% contained residues at levels above the MRL. In mandarins, pesticide residues were most frequently found at levels at or below the MRL (69%), followed by bananas (51%), pears (28%), beans (21%) and potatoes (9%). MRLs were exceeded most often in beans (1.9%), followed by mandarins (1.8%), pears (1.3%), and bananas and potatoes (0.5%). Estimation of the dietary intake of pesticide residues (based on the 90th percentile) from the above-mentioned commodities, where the highest residue levels of the respective pesticides were found, shows that there is no exceeding of the ADI with all the pesticides and commodities studied (European Commission, 1999). In 1998, four commodities (oranges, peaches, carrots, spinach) were analysed for 20 pesticides (acephate, benomyl group, chlopyriphos, chlopyriphos-methyl, deltamethrin, maneb group, diazinon, endosulfan, methamidophos, iprodione, metalaxyl, methidathion, thiabendazole, triazophos, permethrin, vinclozolin, lambdacyalothrin, pirimiphos-methyl, mercabam). With regard to all four commodities investigated in 1998 (oranges, peaches, carrots, spinach), about 32% contained residues of pesticides at or below MRL, and 2% above the MRL (1.8% for EU-MRLs, 0.4% for national MRLs). Residues at or below the MRL were found most often in oranges (67%), followed by peaches (21%), carrots (11%) and spinach (5%). MRL values were exceeded most often in spinach (7.3%), followed by peaches (1.6%), carrots (1.2%)and oranges (0.7%). The intake of pesticide residues has not exceeded the ADI in any case. It was found to be below 10% of the ADI for all pesticides. The exposure ranges from 0.35% of the ADI for the benomyl group to 9.9% of the ADI for the methidathion group. In 1999, four commodities (cauliflower, peppers, wheat grains, and melon) were analysed for the same 20 pesticides as in the 1998 study (European Commission, 2001). Overall, around 4700 samples were analysed. Residues of methamidophos exceeded MRLs most often (8.7%), followed by the maneb group (1.1%), thiabendazole (0.57%), acephate (0.41%) and the benomyl group (0.35%). The MRL for methamidophos was exceeded most often in peppers and melons (18.7 and 3.7%, respectively). The residues of the maneb group exceeded the MRL most often in cauliflower (3.9%); residues of thiabendazole exceeded the MRL most often in melons (2.8% of the melon samples). With regard to all the commodities investigated, around 22% of samples contained residues of pesticides at or below the MRL and 8.7% above the MRL. Residues at or below MRL were found most often in melons (32%), followed by peppers (24%), wheat grains (21%) and cauliflower (17%). MRL values were exceeded most often in peppers (19%), followed by melons (6.1%), cauliflower (3%) and wheat grains (0.5%). The intake of pesticide residues did not exceed the ADI in any case. It was below 1.5% of the ADI for all pesticides. The exposure ranged between 0.43% of the ADI for methamidophos and 1.4% of the ADI for endosulfan. The intakes for the highest residue levels in a composite sample for chlorpyriphos, deltamethrin, endosulfan and methidathion were below the ARfD for adults. They range between 1.5% of the ARfD for deltamethrin and 67% of the ARfD for endosulfan (Nasreddine and Parent-Massin, 2002). In spite of food contamination, most pesticide deaths recorded in hospital surveys are the result of self-poisoning (Eddleston, 2000). The Global Burden of Disease Study 6 estimated that 798 000 people died from deliberate self-harm in 1990, over 75% of whom were from developing countries (Murray and Lopez, 1996). More recent WHO estimates showed that over 500 000 people died from self-harm in Southeast Asia and the Western Pacific during 2000 alone (WHO, 2001). Suicide is the commonest cause of death in young Chinese women and Sri Lankan men and women (Murray and Lopez, 1996; Sri Lankan Ministry of Health, 1995; WHO, 2001).

In India the first report of poisoning due to pesticides was from Kerala in 1958, where over 100 people died after consuming wheat flour contaminated with parathion (Karunakaran, 1958)**.** This prompted the Special Committee on Harmful Effects of Pesticides constituted by the ICAR to focus attention on the problem (Report of the Special Committee of ICAR, 1972). In a multi-centric study to assess the pesticide residues in selected food commodities collected from different states of the country (Surveillance of Food Contaminants in India, 1993), DDT residues were found in about 82% of the 2205 samples of bovine milk collected from 12 states. About 37% of the samples contained DDT residues above the tolerance limit of 0.05 mg/kg (whole milk basis). The highest level of DDT residues found was 2.2 mg/kg. The proportion of the samples with residues above the tolerance limit was highest in Maharastra (74%), followed by Gujarat (70%), Andhra Pradesh (57%), Himachal Pradesh (56%), and Punjab (51%). In the remaining states, this proportion was less than 10%. Data on 186 samples of 20 commercial brands of infants formulae showed the presence of residues of DDT and HCH isomers in about 70 and 94% of the samples with their maximum level of 4.3 and 5.7 mg/kg (fat basis) respectively. Measurement of chemicals in the total diet provides the best estimates of human exposure and of the potential risk. The risk of consumers may then be evaluated by comparison with toxicologically acceptable intake levels. The average total DDT and BHC consumed by an adult were 19.24 mg/day and 77.15 mg/day respectively (Kashyap *et al*., 1994). Fatty food was the main source of these contaminants. In another study, the average daily intake of HCH and DDT by Indians was reported to be 115 and 48 mg per person respectively, which were higher than those observed in most of the developed countries (Kannan *et al*., 1992).

### Impact on environment

Pesticides can contaminate soil, water, turf, and other vegetation. In addition to killing insects or weeds, pesticides can be toxic to a host of other organisms including birds, fish, beneficial insects, and non-target plants. Insecticides are generally the most acutely toxic class of pesticides, but herbicides can also pose risks to non-target organisms.

### Surface water contamination

Pesticides can reach surface water through runoff from treated plants and soil. Contamination of water by pesticides is widespread. The results of a comprehensive set of studies done by the U.S. Geological Survey (USGS) on major river basins across the country in the early to mid- 90s yielded startling results. More than 90 percent of water and fish samples from all streams contained one, or more often, several pesticides (Kole *et al*; 2001). Pesticides were found in all samples from major rivers with mixed agricultural and urban land use influences and 99 percent of samples of urban streams (Bortleson and Davis, 1987–1995). The USGS also found that concentrations of insecticides in urban streams commonly exceeded guidelines for protection of aquatic life (U.S. Geological Survey, 1999). Twenty-three pesticides were detected in waterways in the Puget Sound Basin, including 17 herbicides. According to USGS, more pesticides were detected in urban streams than in agricultural streams (US Department of the Interior, 1995). The herbicides 2,4-D, diuron, and prometon, and the insecticides chlorpyrifos and diazinon, all commonly used by urban homeowners and school districts, were among the 21 pesticides detected most often in surface and ground water across the nation (U.S. Geological Survey, 1998). Trifluralin and 2,4-D were found in water samples collected in 19 out of the 20 river basins studied (Bevans *et al*., 1998; Fenelon *et al*., 1998; Levings *et al*., 1998; Wall *et al*., 1998). The USGS also found that concentrations of insecticides in urban streams commonly exceeded guidelines for protection of aquatic life (U.S. Geological Survey, 1999). According to USGS, “in general more pesticides were detected in urban streams than in agricultural streams”, (Bortleson and Davis, 1987–1995). The herbicide 2,4-D was the most commonly found pesticide, detected in 12 out of 13 streams. The insecticide diazinon, and the weed-killers dichlobenil, diuron, triclopyr, and glyphosate were detected also in Puget Sound basin streams. Both diazinon and diuron were found at levels exceeding concentrations recommended by the National Academy of Sciences for the protection of aquatic life (Bortleson and Davis, 1987–1995).

### Ground water contamination

Groundwater pollution due to pesticides is a worldwide problem. According to the USGS, at least 143 different pesticides and 21 transformation products have been found in ground water, including pesticides from every major chemical class. Over the past two decades, detections have been found in the ground water of more than 43 states (Waskom, 1994). During one survey in India, 58% of drinking water samples drawn from various hand pumps and wells around Bhopal were contaminated with Organo Chlorine pesticides above the EPA standards (Kole and Bagchi, 1995). Once ground water is polluted with toxic chemicals, it may take many years for the contamination to dissipate or be cleaned up. Cleanup may also be very costly and complex, if not impossible (Waskom 1994; O'Neil, 1998; US EPA, 2001).

### Soil contamination

A large number of transformation products (TPs) from a wide range of pesticides have been documented (Barcelo' and Hennion, 1997; Roberts, 1998; Roberts and Hutson, 1999). Not many of all possible pesticide TPs have been monitored in soil, showing that there is a pressing need for more studies in this field. Persistency and movement of these pesticides and their TPs are determined by some parameters, such as water solubility, soil-sorption constant (K_oc_), the octanol/water partition coefficient (K_ow_), and half-life in soil (DT_50_). Pesticides and TPs could be grouped into:(a) Hydrophobic, persistent, and bioaccumulable pesticides that are strongly bound to soil. Pesticides that exhibit such behavior include the organochlorine DDT, endosulfan, endrin, heptachlor, lindane and their TPs. Most of them are now banned in agriculture but their residues are still present. (b) Polar pesticides are represented mainly by herbicides but they include also carbamates, fungicides and some organophosphorus insecticide TPs. They can be moved from soil by runoff and leaching, thereby constituting a problem for the supply of drinking water to the population. The most researched pesticide TPs in soil are undoubtedly those from herbicides. Several metabolic pathways have been suggested, involving transformation through hydrolysis, methylation, and ring cleavage that produce several toxic phenolic compounds. The pesticides and their TPs are retained by soils to different degrees, depending on the interactions between soil and pesticide properties. The most influential soil characteristic is the organic matter content. The larger the organic matter content, the greater the adsorption of pesticides and TPs. The capacity of the soil to hold positively charged ions in an exchangeable form is important with paraquat and other pesticides that are positively charged. Strong mineral acid is required for extracting these chemicals, without any analytical improvement or study reported in recent years. Soil pH is also of some importance. Adsorption increases with decreasing soil pH for ionizable pesticides (e.g. 2,4-D,2,4,5-T, picloram, and atrazine) (Andreu and Pico', 2004).

### Effect on soil fertility (beneficial soil microorganisms)

Heavy treatment of soil with pesticides can cause populations of beneficial soil microorganisms to decline. According to the soil scientist Dr. Elaine Ingham, “If we lose both bacteria and fungi, then the soil degrades. Overuse of chemical fertilizers and pesticides have effects on the soil organisms that are similar to human overuse of antibiotics. Indiscriminate use of chemicals might work for a few years, but after awhile, there aren't enough beneficial soil organisms to hold onto the nutrients” (Savonen, 1997). For example, plants depend on a variety of soil microorganisms to transform atmospheric nitrogen into nitrates, which plants can use. Common landscape herbicides disrupt this process: triclopyr inhibits soil bacteria that transform ammonia into nitrite (Pell *et al*., 1998); glyphosate reduces the growth and activity of free-living nitrogen-fixing bacteria in soil (Santos and Flores, 1995) and 2,4-D reduces nitrogen fixation by the bacteria that live on the roots of bean plants (Arias and Fabra, 1993; Fabra et al., 1997), reduces the growth and activity of nitrogen-fixing blue-green algae (Singh and Singh, 1989; Tözüm-Çalgan and Sivaci-Güner, 1993), and inhibits the transformation of ammonia into nitrates by soil bacteria (Frankenberger *et al*., 1991, Martens and Bremner, 1993). Mycorrhizal fungi grow with the roots of many plants and aid in nutrient uptake. These fungi can also be damaged by herbicides in the soil. One study found that oryzalin and trifluralin both inhibited the growth of certain species of mycorrhizal fungi (Kelley and South, 1978). Roundup has been shown to be toxic to mycorrhizal fungi in laboratory studies, and some damaging effects were seen at concentrations lower than those found in soil following typical applications (Chakravarty and Sidhu, 1987; Estok *et al*., 1989). Triclopyr was also found to be toxic to several species of mycorrhizal fungi (Chakravarty and Sidhu, 1987) and oxadiazon reduced the number of mycorrhizal fungal spores (Moorman, 1989).

### Contamination of air, soil, and non-target vegetation

Pesticide sprays can directly hit non-target vegetation, or can drift or volatilize from the treated area and contaminate air, soil, and non-target plants. Some pesticide drift occurs during every application, even from ground equipment (Glotfelty and Schomburg, 1989). Drift can account for a loss of 2 to 25% of the chemical being applied, which can spread over a distance of a few yards to several hundred miles. As much as 80–90% of an applied pesticide can be volatilised within a few days of application (Majewski, 1995). Despite the fact that only limited research has been done on the topic, studies consistently find pesticide residues in air. According to the USGS, pesticides have been detected in the atmosphere in all sampled areas of the USA (Savonen, 1997). Nearly every pesticide investigated has been detected in rain, air, fog, or snow across the nation at different times of the year (U.S. Geological Survey, 1999). Many pesticides have been detected in air at more than half the sites sampled nationwide. Herbicides are designed to kill plants, so it is not surprising that they can injure or kill desirable species if they are applied directly to such plants, or if they drift or volatilise onto them. Many ester-formulation herbicides have been shown to volatilise off treated plants with vapors sufficient to cause severe damage to other plants (Straathoff, 1986). In addition to killing non-target plants outright, pesticide exposure can cause sublethal effects on plants. Phenoxy herbicides, including 2,4-D, can injure nearby trees and shrubs if they drift or volatilise onto leaves (Dreistadt *et al*., 1994). Exposure to the herbicide glyphosate can severely reduce seed quality (Locke *et al*., 1995). It can also increase the susceptibility of certain plants to disease (Brammall and Higgins, 1998). This poses a special threat to endangered plant species. The U.S. Fish and Wildlife Service has recognized 74 endangered plants that may be threatened by glyphosate alone (U.S. EPA Office of Pesticides and Toxic Substances, 1986). Exposure to the herbicide clopyralid can reduce yields in potato plants (Lucas and Lobb, 1987). EPA calculated that volatilisation of only 1% of applied clopyralid is enough to damage non-target plants (US EPA, 1990). Some insecticides and fungicides can also damage plants (Dreistadt *et al*., 1994). Pesticide damage to plants is commonly reported to state agencies in the Northwest. (Oregon Dept. of Agriculture, 1999; Washington Dept. of Health, 1999). Plants can also suffer indirect consequences of pesticide applications when harm is done to soil microorganisms and beneficial insects. Pesticides including those of new the generation, e.g., dacthal, chlorothalonil, chlorpyrifos, metolachlor, terbufos and trifluralin have been detected in Arctic environmental samples (air, fog, water, snow) (Rice and Cherniak, 1997), and (Garbarino *et al*., 2002). Other studies have identified the ability of some of these compounds to undergo short-range atmospheric transport (Muir *et al*., 2004) to ecologically sensitive regions such as the Chesapeake Bay and the Sierra Nevada mountains (LeNoir *et al*., 1999; McConnell *et al*., 1997; Harman-Fetcho *et al*., 2000, Thurman and Cromwell , 2000). One long-term study that investigated pesticides in the atmosphere of British Columbia (BC), dating from 1996 (Belzer *et al*., 1998) showed that 57 chemicals were investigated at two sampling sites (Agassiz and Abbotsford) in the Fraser Valley, from February 1996 until March 1997. Atrazine, malathion, and diazinon, highly toxic chemicals identified as high-priority pesticides by Verrin *et al*. (2004), were detected as early as the end of February (72 pg/m^3^) until mid-October (253 pg/m^3^), with a peak concentration in mid-June of 42.7 ngm^−3^. Dichlorvos is a decomposition product of another pesticide, Naled (Dibrom) (Hall *et al*., 1997). Captan and 2,4-D showed the highest concentrations and deposition rates at these two sites, followed by dichlorvos and diazinon (Dosman and Cockcraft, 1989). Air concentrations of currently used pesticides in Alberta were investigated in 1999 at four sampling sites that were chosen according to geography and pesticide sales data (Kumar, 2001). Triallate and trifluralin were the two mostly detected pesticides at the four sites. Insecticides (malathion, chlorpyrifos, diazinon and endosulfan) were detected intermittently with concentrations in the range 20–780 pg/m^3^. South of Regina, Saskatchewan, in 1989 and 1990, 2,4-D reached 3.9 and 3.6 ng/m^3^ at the end of June (Waite *et al*., 2002a). Triallate, dicamba, bromoxynil concentrations were also higher in 1989 (peak concentration of 4.2 ng/m^3^ in mid-June) compared with 1990 (600–700 pg/m^3^ in mid-June). In a more recent study, Waite et al. (2005) studied spatial variations of selected herbicides on a threesite, 500km transect that included two agricultural sites—Bratt's Lake, located 35 km southwest of Regina and Hafford to the North—and a background site at Waskesiu. Some acid herbicides were also investigated in South Tobacco Creek, Manitoba during 1993–1996. Once again, maximum concentrations occurred during periods of local use (Rawn *et al*., 1999a). A neutral herbicide, atrazine, was also investigated in 1995 (Rawn *et al*., 1998). It was first detected in mid-April, peaked mid- June at about 300 pg/m^3^, and was detected until the end of October. The insecticide dacthal was identified throughout the sampling periods in 1994, 1995 and 1996 (Rawn and Muir, 1999) even though it was not used in this area (<20–300 pg/m^3^).

### Non-target organisms

Pesticides are found as common contaminants in soil, air, water and on non-target organisms in our urban landscapes. Once there, they can harm plants and animals ranging from beneficial soil microorganisms and insects, non-target plants, fish, birds, and other wildlife. Chlorpyrifos, a common contaminant of urban streams (U.S. Geological Survey, 1999), is highly toxic to fish, and has caused fish, kills in waterways near treated fields or buildings (US EPA, 2000). Herbicides can also be toxic to fish. According to the EPA, studies show that trifluralin, an active ingredient in the weed-killer Snapshot, “is highly to very highly toxic to both cold and warm water fish” (U.S. EPA, 1996). In a series of different tests it was also shown to cause vertebral deformities in fish (Koyama, 1996). The weed-killers Ronstar and Roundup are also acutely toxic to fish (Folmar *et al*., 1979; Shafiei and Costa, 1990). The toxicity of Roundup is likely due to the high toxicity of one of the inert ingredients of the product (Folmar *et al*., 1979). In addition to direct acute toxicity, some herbicides may produce sublethal effects on fish that lessen their chances for survival and threaten the population as a whole. Glyphosate or glyphosate-containing products can cause sublethal effects such as erratic swimming and labored breathing, which increase the fish's chance of being eaten (Liong *et al*., 1988). 2,4-D herbicides caused physiological stress responses in sockeye salmon (McBride et al., 1981) and reduced the food-gathering abilities of rainbow trout (Little, 1990). Several cases of pesticide poisoning of dolphins have been reported worldwide. Because of their high trophic level in the food chain and relatively low activities of drug-metabolising enzymes, aquatic mammals such as dolphins accumulate increased concentrations of persistent organic pollutants (Tanabe *et al*., 1988) and are thereby vulnerable to toxic effects from contaminant exposures. Dolphins inhabiting riverine and estuarine ecosystems are particularly vulnerable to the activities of humans because of the restricted confines of their habitat, which is in close proximity to point sources of pollution. River dolphins are among the world's most seriously endangered species. Populations of river dolphins have been dwindling and face the threat of extinction; the Yangtze river dolphin (Lipotes vexillifer) in China and the Indus river dolphin (Platanista minor) in Pakistan are already close to extinction (Renjun, 1990; Perrin *et al*., 1989; Reeves *et al*., 1991; Reeves and Chaudhry, 1998). In addition to habitat degradation (such as construction of dams) (Reeves and Leatherwood, 1994), boat traffic, fishing, incidental and intentional killings, and chemical pollution have been threats to the health of river dolphins (Kannan *et al*., 1993b, 1994, 1997; Senthilkumar *et al*., 1999). Earlier studies reported concentrations of heavy metals (Kannan *et al*., 1993), organochlorine pesticides and polychlorinated biphenyls (PCBs) (Kannan *et al*., 1994), and butyltin compounds (Kannan *et al*., 1997) in Ganges river dolphins and their prey. The continuing use of organochlorine pesticides and PCBs in India is of concern (Kannan *et al*., 1992; Kannan *et al*., 1997a; Kannan *et al*., 1997b; Tanabe *et al*., 1998). The Ganges river basin is densely populated and heavily polluted by fertilizers, pesticides, and industrial and domestic effluents (Mohan, 1989). In addition to fish, other marine or freshwater animals are endangered by pesticide contamination. Exposure to great concentrations of persistent, bioaccumulative, and toxic contaminants such as DDT (1,1,1-trichloro-2,2-bis[*p*-chlorophenyl]ethane) and PCBs has been shown to elicit adverse effects on reproductive and immunological functions in captive or wild aquatic mammals (Helle *et al*., 1976; Reijnders, 1986; Ross *et al*., 1995; Martineau *et al*., 1987; Kannan *et al*., 1993; Colborn and Smolen, 1996). Aquatic mammals inhabiting freshwater systems, such as otters and mink, have been reported to be sensitive to chemical contamination (Leonards *et al*., 1995; Leonards *et al*., 1997). 2,4-D or 2,4-D containing products have been shown to be harmful to shellfish (Cheney *et al*., 1997) and other aquatic species (U.S. EPA, 1989; Sanders, 1989) The weed-killer trifluralin is moderately to highly toxic to aquatic invertebrates, and highly toxic to estuarine and marine organisms like shrimp and mussels (U.S. EPA, 1996). Since herbicides are designed to kill plants, it makes sense that herbicide contamination of water could have devastating effects on aquatic plants. In one study, oxadiazon was found to severely reduce algae growth (Ambrosi *et al*., 1978). Algae is a staple organism in the food chain of aquatic ecosystems. Studies looking at the impacts of the herbicides atrazine and alachlor on algae and diatoms in streams showed that even at fairly low levels, the chemicals damaged cells, blocked photosynthesis, and stunted growth in varying ways (U.S. Water News Online, 2000). The herbicide oxadiazon is also toxic to bees, which are pollinators (Washington State Department of Transportation, 1993). Herbicides may hurt insects or spiders also indirectly when they destroy the foliage that these animals need for food and shelter. For example spider and carabid beetle populations declined when 2,4-D applications destroyed their natural habitat (Asteraki *et al*., 1992). Non-target birds may also be killed if they ingest poisoned grains set out as bait for pigeons and rodents (US EPA, 1998). Avitrol, a commonly used pigeon bait, poses a large potential for ingestion by non target grain feeding birds. It can be lethal to small seed-eating birds (Extoxnet, 1996). Brodifacoum, a common rodenticide, is highly toxic to birds. It also poses a secondary poisoning hazard to birds that may feed on poisoned rodents (US EPA, 1998). Herbicides can also be toxic to birds. Although trifluralin was considered “practically nontoxic to birds” in studies of acute toxicity, birds exposed multiple times to the herbicide experienced diminished reproductive success in the form of cracked eggs (U.S. EPA, 1996). Exposure of eggs to 2,4-D reduced successful hatching of chicken eggs (Duffard *et al*., 1981) and caused feminisation or sterility in pheasant chicks (Lutz *et al*., 1972). Herbicides can also adversely affect birds by destroying their habitat. Glyphosate treatment in clear cuts caused dramatic decreases in the populations of birds that lived there (MacKinnon *et al*., 1993) Effects of some organochlorines (OCs) on fish-eating water birds and marine mammals have been documented in North America and Europe (Barron *et al*., 1995; Cooke, 1979; Kubiak *et al*., 1989). Despite the continuing usage, little is known about the impacts of OCs in bird populations in developing countries. Among the countries that continue to use OCs, India has been one of the major producers and consumers in recent years. As a consequence, wild birds in India are exposed to great amounts of OC pesticides (Tanabe *et al*., 1998). Use of OCs in tropical countries may not only result in exposure of resident birds but also of migratory birds when they visit tropical regions in winter. The Indian sub-continent is a host to a multitude of birds from western Asia, Europe and Arctic Russia in winter(Woodcock, 1980). Hundreds of species of waterfowl, including wading birds such as plovers, terns and sandpipers, migrate each winter to India covering long distances (Grewal, 1990). While concentrations of OC pesticides in wholebody homogenates of birds have been reported elsewhere (Tanabe *et al*., 1998), concentrations of OCs in prey items and in eggs of Indian birds have not been reported.

A few studies related to the decline in the populations of bats in various parts of the world to OC exposure were also being conducted (Altenbach *et al*., 1979; Clark, 1976; Clark, 1983; Clark, 1981; Geluso *et al*., 1976; Jefferies, 1976; Thies and Mc Bee, 1994). The world population of bats was estimated to be 8.7 million during 1936 and it declined to approximately 200,000 in 1973 (Geluso *et al*., 1976) It has recovered slightly to an estimated number of 700,000 in 1991 (Geluso *et al*., 1976; Thies and Mc Bee, 1994). High tissue concentrations of *p,p'*-dichlorodiphenyldichloroethene (*p,p'*–DDE) have been found in bats in Carlsbad Caverns in Mexico and in New Mexico in the USA (Geluso *et al*., 1976; Thies and Mc Bee, 1994). Occurrence of stillbirths in little brown bats exposed to high concentrations of PCBs, *p,p'*–DDE, and/or oxychlordane was documented (Clark, 1976; Jefferies, 1976). These observations indicate that bats can accumulate high concentrations of OCs and may be affected by their potential toxic effects. The flying fox or the new world fruit bat, short-nosed fruit bat and Indian pipistrelle bat are resident species and are very common in South India. Their habitat is mainly agricultural areas, rock caves, and abandoned houses in domesticated areas. Insects constitute an important diet for many bats, allowing the passage of OCs in their body (Mc Bee *et al*., 1992). Several studies found OC pesticides and PCBs in livers and eggs of birds in developed countries (Becker, 1989; Bernardz *et al*., 1990; Cade *et al*., 1989; Castillo *et al*., 1994; Mora, 1996; Mora, 1997). Similarly, several studies reported OCs in a variety of biota including humans and wildlife from India (Senthilkumar *et al*., 2000). However, no study has used whole body homogenates of birds, which is important to evaluate biomagnification features and body burdens of OCs (Mc Bee *et al*., 1992). Earlier studies used specific body tissues to estimate biomagnification of OCs. However theoretically, estimation of biomagnification factors requires whole body concentrations rather than specific tissue concentrations.

## Conclusion

The data on environmental-cum-health risk assessment studies may be regarded as an aid towards a better understanding of the problem. Data on the occurrence of pesticide-related illnesses among defined populations in developing countries are scanty. Generation of base-line descriptive epidemiological data based on area profiles, development of intervention strategies designed to lower the incidence of acute poisoning and periodic surveillance studies on high risk groups are needed. Our efforts should include investigations of outbreaks and accidental exposure to pesticides, correlation studies, cohort analyses, prospective studies and randomised trials of intervention procedures. Valuable information can be collected by monitoring the end product of human exposure in the form of residue levels in body fluids and tissues of the general population. The importance of education and training of workers as a major vehicle to ensure a safe use of pesticides is being increasingly recognised.

Because of the extensive benefits which man accrues from pesticides, these chemicals provide the best opportunity to those who juggle with the risk-benefit equations. The economic impact of pesticides in non-target species (including humans) has been estimated at approximately $8 billion annually in developing countries. What is required is to weigh all the risks against the benefits to ensure a maximum margin of safety. The total cost-benefit picture from pesticide use differs appreciably between developed and developing countries. For developing countries it is imperative to use pesticides, as no one would prefer famine and communicable diseases like malaria. It may thus be expedient to accept a reasonable degree of risk. Our approach to the use of pesticides should be pragmatic. In other words, all activities concerning pesticides should be based on scientific judgement and not on commercial considerations. There are some inherent difficulties in fully evaluating the risks to human health due to pesticides. For example there is a large number of human variables such as age, sex, race, socio-economic status, diet, state of health, *etc.* – all of which affect human exposure to pesticides. But practically little is known about the effects of these variables. The long-term effects of low level exposure to one pesticide are greatly influenced by concomitant exposure to other pesticides as well as to pollutants present in air, water, food and drugs.

Pesticides are often considered a quick, easy, and inexpensive solution for controlling weeds and insect pests in urban landscapes. However, pesticide use comes at a significant cost. Pesticides have contaminated almost every part of our environment. Pesticide residues are found in soil and air, and in surface and ground water across the countries, and urban pesticide uses contribute to the problem. Pesticide contamination poses significant risks to the environment and non-target organisms ranging from beneficial soil microorganisms, to insects, plants, fish, and birds. Contrary to common misconceptions, even herbicides can cause harm to the environment. In fact, weed killers can be especially problematic because they are used in relatively large volumes. The best way to reduce pesticide contamination (and the harm it causes) in our environment is for all of us to do our part to use safer, non-chemical pest control (including weed control) methods.

The exercise of analysing the range and nature of benefits arising from pesticide use has been a mixture of delving, dreaming and distillation. There have been blind alleys, but also positive surprises. The general picture is as we suspected: there is publicity, ideological kudos and scientific opportunity associated with ‘knocking’ pesticides, while praising them brings accusations of vested interests. This is reflected in the imbalance in the number of published scientific papers, reports, newspaper articles and websites against and for pesticides. The colour coding for types of benefit, economic, social or environmental, reveals the fact that at community level, most of the benefits are social, with some compelling economic benefits. At national level, the benefits are principally economic, with some social benefits and one or two issues of environmental benefits. It is only at global level that the environmental benefits really come into play.

There is a need to convey the message that prevention of adverse health effects and promotion of health are profitable investments for employers and employees as a support to a sustainable development of economics. To sum up, based on our limited knowledge of direct and/or inferential information, the domain of pesticides illustrates a certain ambiguity in situations in which people are undergoing life-long exposure. There is thus every reason to develop health education packages based on knowledge, aptitude and practices and to disseminate them within the community in order to minimise human exposure to pesticides.
